# Antibacterial Efficiency of three Different Irrigation Methods in Infected Roots Infected with *Enterococcus Faecalis* Biofilm

**DOI:** 10.30476/dentjods.2025.102497.2365

**Published:** 2025-09-01

**Authors:** Maryam Zare Jahromi, Ali Baghersad, Amir Mansour Shirani, Arezoo Tahmourespour, Elham Alipour, Amirreza Mokabberi

**Affiliations:** 1 Dept. of Endodontics, School of Dentistry, Isfahan (Khorasgan) Branch, Islamic Azad University, Isfahan, Iran.; 2 Dentist, School of Dentistry, Isfahan (Khorasgan) Branch, Islamic Azad University, Isfahan, Iran.; 3 Dept. of Oral Medicine, Dental School, Isfahan (Khorasgan) Branch, Islamic Azad University, Isfahan, Iran.; 4 Dept. of Basic Medical Sciences, School of Dentistry, Isfahan (Khorasgan) Branch, Islamic Azad University, Isfahan, Iran.; 5 Postgraduate Student, Dept. of Operative Dentistry, Faculty of Dentistry, Isfahan (Khorasgan) Branch, Islamic Azad University, Isfahan, Iran.; 6 Postgraduate Student, Dept. of Endodontics, School of Dentistry, Isfahan (Khorasgan) Branch, Islamic Azad University, Isfahan, Iran.

**Keywords:** Diode Lasers, *Enterococcus Faecalis*, Root Canal, Antibacterial

## Abstract

**Background:**

The elimination of pathogenic microorganisms is crucial in endodontic treatments, as *Enterococcus Faecalis* is involved in the majority of endodontic failures. This bacterium is known for its resilience and ability to persist within the root canal system, often leading to treatment complications.

**Purpose:**

The aim of this study was to compare the antibacterial efficiency of three different irrigation methods including passive ultrasonic, XP Endofinisher file, and Laser Diode 810 nm in infected roots with *Enterococcus Faecalis* (*E.faecalis*) biofilm.

**Materials and Method:**

In this experimental study, 48 anterior single-canal teeth were enrolled. After cutting their crowns, the teeth were cultured with *E.faecalis* and then randomly divided into four groups. Following preparation through the rotary system up to F4 at the working length, passive ultrasonic irrigation (Ultra X) was used inside the root canal in the first group. In the second group of the study, the XP Endofinisher file was applied to activate the irrigation solution, while in the third group, the Laser Diode 810 nm was used. The fourth group served as the control group and did not utilize any irrigation. The irrigation solution employed across all groups consisted of 1ml of 5.25% sodium hypochlorite (NaOCl), followed by a final irrigation with 5ml of 17% ethylenediaminetetraacetic acid (EDTA), 5ml of 5.25% NaOCl, and 5ml of sterile saline. After canal irrigation and sampling, bacterial colony counting was conducted, and the data were recorded. If the data were normally distributed, a variance test analysis was used; otherwise, the non-parametric Kruskal-Wallis test was applied. The tests were performed at a 5% significance level using SPSS software version 24.

**Results:**

The reduction in the number of bacterial colonies was significantly greater in all three methods compared to the control group. The obtained data revealed that the antibacterial effect of Laser 810 nm was considerably (*p*< 0.05) higher than the other two groups and reduction in the number of colonies in Ultra X group was remarkably (*p*< 0.05) greater than the XP Endofinisher file group.

**Conclusion:**

All three mentioned methods were effective in reducing the number of bacteria in endodontic treatments. Notably, the antibacterial efficiency of the Laser Diode 810 nm was significantly greater than that of the other two methods.

## Introduction

The principal purpose of endodontic treatments is to minimize or eliminate microorganisms from the infected root canal which can be achieved by canal shaping and using different irrigation methods [ 1 - 2 ]. *Enterococcus Faecalis* (*E. faecalis*) is an anaerobic gram-positive coccus that is usually present in the human oral cavity which becomes asymptomatic in persistent endodontic infections due to its good adaptation in rich nutrient and low oxygen levels environment [ 3 - 4 ].

Irrigation methods play a crucial role in root canal treatment by performing significant mechanical, chemical, and microbiological functions. These functions allow for effective cleaning of areas that are not accessible to mechanical instruments, addressing the complexities of root canal systems, which often include lateral canals, isthmuses, and deltas. Consequently, achieving complete cleaning of the root canal can be challenging due to these structural complications [ 5 ]. Sodium hypochlorite (NaOCl) is a common irrigant solution that can be used alone or in combination with chlorhexidine and/or with ethylenediaminetetraacetic acid (EDTA) in endodontics treatments and it is recognized as the most effective tissue solvent and antimicrobial agent in root canal treatment due to its ability to form hypochlorous acid when it comes into contact with organic debris. This reaction enhances its antibacterial activity, making it a critical component in eliminating bacteria within the root canal system [ 6 ]. Moreover, NaOCl has an indispensable role in chemical preparation of root canal treatment and could be affected by different parameters such as concentration, temperature, exposure time, and pH value of the irrigant. Several techniques have evolved to activate NaOCl, as the use of it alone can be ineffective during chemical preparation [ 7 ].

Different techniques are used to irrigate inside the root canal, one of these methods is using an ultrasonic instrument, which was first introduced by Richman at a frequency of 25 to 30 kHz [ 8 ]. In accordance with previous studies, two types of irrigation methods using ultrasonic instruments have been described: ultrasonic irrigation (UI) and passive ultrasonic irrigation (PUI). In UI, the ultrasonic file is placed in direct contact with the canal walls. However, this method has been shown to be less effective in eliminating pulp tissue, removing the smear layer, and achieving root canal disinfection compared to PUI. Clearly, in the PUI method, after the preparation of the canal up to the master apical file, a small file (such as size 15) enters the root canal and is placed at a distance from the apical area, then the canal is irrigated through the transmission of energy and vibration to irrigation agent by the file [ 8-10 ].

One of the newest technologies in irrigation by ultrasonic system is the Ultra X device from Eighteeth Co. Di Nardo *et al*. [ 11 ] represented a study on the Ultra X device and reported that this instrument is more effective in removing debris specifically in lateral canals rather than sonic Endoactivator system. In recent years, the XP Endofinisher file has emerged as a significant technique in endodontic treatments. This file serves as an adjunctive approach aimed at enhancing the effectiveness of irrigation and disinfection during procedures. It has shown potential as an additional therapeutic tool to maximize bacterial removal from root canals [ 12 ]. The innovative design of the XP Endofinisher files significantly enhances cleaning in root canals while preserving dentin, allowing access to areas of the canal that were previously difficult to reach [ 13 ]. According to Bao *et al*. [ 14 ], the XP Endofinisher files have been shown to effectively remove biofilm from the apical part of the canal, proving particularly beneficial in challenging areas.

Another effective method that has been identified in endodontic processes is the utilization of lasers to irrigate inside root canal. The evidence represented the effectiveness of lasers in removing the smear layer and disinfecting the root canal during endodontic treatments [ 15 ]. One of the notable benefits of laser techniques in dentistry is their ability to minimize dentin erosion [ 6 ]. Diode lasers, specifically, can be effectively used for photothermal disinfection and the activation of irrigants, such as NaOCl and EDTA, in endodontic treatments. The diode laser technique offers significant advantages in endodontics, particularly in root canal disinfection. One of its key benefits is its small size and flexible, thin fiber, which allows for easy access to narrow canals. This feature enhances the efficacy of disinfection in the radicular dentinal tubules, making it an effective tool for treating complex root canal systems [ 16 ]. 

This study focuses on the critical role of removing pathogenic microorganisms, particularly *E. faecalis*, in endodontic treatments to prevent failures. It aims to compare three irrigation methods: PUI, XP Endofinisher file, and Laser Diode 810 nm, assessing their effectiveness in eliminating bacteria from the root canal system. 

## Materials and Method

### Selection and Description of Participants

### Tooth preparation procedure

After approval by Ethics and Research committee of Islamic Azad University of Khorasgan Branch (IR.IAU. KHUISF.REC.1402.010), 48 anterior single-canal teeth, extracted for periodontal reasons were enrolled in this study. The inclusion criteria were single canal teeth, without severe curvature, no calcification, mature teeth with closed apex and root without cracks or fractures. The teeth were evaluated using radiography with a parallel technique, and those that were free from caries, internal and external root resorption, and restorations were selected. After debriding the surface of each root with a curette, the samples were immersed in 5.25% NaOCl solution (Morvabon, Iran) for 1h and kept in sterile saline until preparation. 

Firstly, the crowns of the teeth were cut from the cemento-enamel junction (CEJ) using a diamond bur to achieve a standard root length of 15 mm. A K-file #15 (Mani, Inc., Japan) was then used to determine the length, with 0.5 mm subtracted from this measurement to establish the working length. Subsequently, the canals of the studied teeth were prepared using the crown-down technique with ProTaper Universal rotary files (Dentsply Maillefer, Switzerland), following the manufacturer's instructions up to size F2 (Sx, S1, S2, F1, and F2). Initially, the orifice of the canal was enlarged using an Sx file. Then, shaping files (S1 and S2) were utilized with a brushing action during the withdrawal stroke to create a straight-line radicular access and to passively progress apically. After that, finishing files (F1 and F2) were used passively to reach the working length. During cleaning and shaping between each file number, irrigation with 3 ml of 5.25% NaOCl was employed with the help of an irrigation syringe (Supa Co, Tehran, Iran) with a side vented needle gauge 30 (Tribest, China). For the final irrigation process, 3ml of 17% EDTA solution was applied for 1 minute. Additionally, 3ml of 5.25% NaOCl and 3ml of sterile saline were also used. Subsequently, the canal was dried through the sterile paper cone (Meta, South Korea) and the apex was sealed with cyanoacrylate, then the surface of the roots was covered with two layers of nail polish. Each tooth was transferred to a test tube containing sterile brain heart infusion (BHI-broth) culture medium and autoclaved at 121°C and 15Pa pressure for 30 minutes. For accuracy of the sterilization process, five teeth were randomly selected and incubated for 24 hours at 37 degrees in BHI-broth. The aforementioned teeth were not included in the study groups; the absence of bacterial growth indicated the absence of contamination.

Frozen *E. faecalis* bacterium (ATCC 9854) was transferred to BHI agar plates (Merck, KGaA, Germany) and incubated for 24 h at 37°C under anaerobic conditions with 5% CO_2_. Then, 0.5 Mcfarland standard concentration of broth including 1.5×10^8^ CFUs/ml was prepared. 100μl of this suspension was injected into each canal through the sterile insulin syringe. The canals were then incubated under anaerobic conditions at 37°C for the formation of *E. faecalis* biofilm on the root surface for 3 weeks. 

In all groups, the remained BHI-broth culture medium in the canal was first dried with a sterile paper cone. Afterward, the preparation of the canals, which was done in the previous step up to the F2 rotary file and the process continued until F4. It should be mentioned that the irrigation process was done between each F3 and F4 rotary files. The irrigation agent used in all studied groups except control group was 1 ml of 5.25% NaOCl between each file number, and the final irrigation agent was 5ml of 17% EDTA, 5ml of 5.25% NaOCl and 5ml of sterile saline. 

### Data Collection and Measurements

### Passive Ultrasonic Group

The irrigation process of this group performed through the means of an Ultra X instrument (Eighteeth, Changzhou Sifary Medical Technology Co, Ltd, Changzhou City, China). After final preparation up to F4, irrigation was done between changing each file number by using an Ultra X devise inside the canal at 1mm from WL and moved 2-3 mm vertically up and down for 60 seconds at high power set with a blue ultrasonic tip [ 17 ]. 

### XP Endofinisher Group

In this group as in the previous group, the preparation and irrigation were conducted. Then, the XP Endofinisher file (FKG, Switzerland) was placed 1mm shorter than the working length by endomotor. Then, a rotating movement with a frequency of 800 rpm for 1 minute in a range of 1 or 2mm up and down movements (according to the manufacturer's instructions) was applied gently and slowly [ 17 ].

### Diode laser 810 nm Group 

After the irrigation procedure of this group by Laser Diode 810 nm (Nita Co, China), the canal was finally dried through the sterile paper cone No #40. The laser was performed by a flexible 200μ optical fiber in the 810 nm wavelength with a power of 1.5 W. The fiber was 1mm shorter than the working length and the device was used in continuous wave mode. Each tooth was subjected to two radiation cycles. The laser handpiece was held at a 10-degree angle to the longitudinal axis of the tooth and applied circularly in an apical to coronal direction without water or any type of cooling.

### Negative control group

The negative control group did not receive any irrigation or activation agents. This group represented the initial number of bacteria and was used as a standard for comparison.

### Sampling

Microbial sampling was done from inside the canals by piezorimer (No. 4) to transfer dentin chips to tubes containing 1 ml of saline. After that, three sterile paper cones No #40 were used to transfer the remaining liquid to the tubes. Immersion was done for 20s and then 10 dilutions were prepared from each tube. 100μl of each dilution were transferred to BHI-agar plates, cultured for 48 h at 37°C and were incubated in 5% CO_2_. Finally, *E. faecalis* colonies were counted based on CFU/ml. 

### Statistics

The analysis was done at two descriptive and inferential levels. At the descriptive level of qualitative variables, frequency indicators and frequency percentage, and in quantitative variables, average indicators, and standard deviation were reported. At the inferential level, if the data were normally distributed, an analysis of variance (ANO-VA) test was used; otherwise, the non-parametric Kruskal-Wallis test was applied. The tests were performed at a five percent error level using SPSS soft-ware version 24. 

## Results

### Descriptive analysis

**Figure 1:**
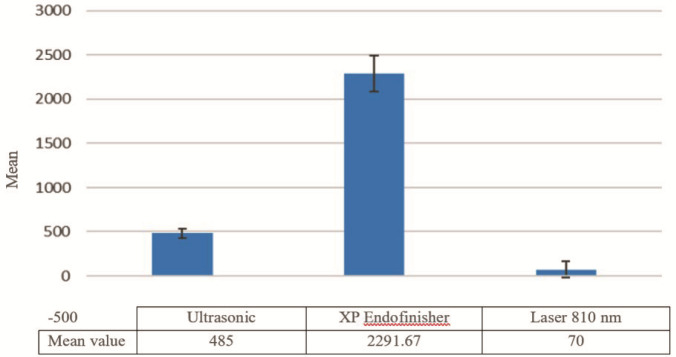
The average antibacterial effect by calculating the bacterial colonies in terms of colony-forming unit per millilitre (CFU/ml) in three irrigation techniques by using the ultrasonic, XP finisher, and laser methods

Table 1 summarizes a descriptive statistic of the antibacterial effect using the Ultrasonic, XP Endofinisher file, and Laser method with the control group. The mean value of antibacterial efficiency in the ultrasonic irrigation method was obtained 485, while in the case of XP Endofinisher file and Laser Diode 810 nm; the mean values were measured to be 2291.67 and 70, respectively. Also, the comparison of the average antibacterial efficiency of these three irrigation methods is represented in Figure 1.

**Table 1:** Descriptive statistics of average antibacterial effect along with calculating the bacterial population based on colony-forming unit per millilitre (CFU/ml) in ultrasonic, XP Endofinisher, laser irrigation method and control group

Method	Number	Minimum	Maximum	Mean	Standard deviation
Ultrasonic	12	420	620	485.00	53.343
XP Endofinisher	12	2000	2600	2291.67	202.072
Laser	12	0	280	70.00	94.099
Control	12	2000000	10000000	4733333.3	2250387.172

### Inferential analysis

In this section, the hypothesis of the research, which was to compare the mean antibacterial effect of three irrigation methods- Ultrasonic, XP Endofinisher file, and laser- has been investigated. It should be mentioned that the normality of variables was assessed using the Shapiro-Wilk test, and the obtained values are presented in Table 2.

**Table 2:** Investigating the normality of the antibacterial effect in Ultrasonic, XP Endofinisher, and Laser irrigation methods

Variable	Degrees of freedom	Test statistic	Significance level
Ultrasonic	12	0.875	0.076
XP Endofinisher	12	0.937	0.456
Laser	12	0.715	0.001*
Control	12	0.831	0.002*

*Significance level 0.05

According to the Shapiro-Wilk test, the antibacterial effect of the Ultrasonic and XP Endofinisher files had a significance level of 0.076 and 0.456, respectively (*p*> 0.05). Therefore, at an error level of 5%, there was no significant difference between the distribution of variables and normal distribution, while in the case of Laser Diode 810nm and control groups; the antibacterial effect has reported a significance level of 0.001 and 0.022, respectively. It can be deduced that, at the error level of 5%, these variables did not follow the normal distribution (Table 2).

Therefore, according to the results from Table 2, we used the Kruskal-Wallis test to evaluate the research hypothesis. According to the data presented in Table 3, the significance level of the Kruskal-Wallis test was less than 0.001. This indicates that, at a 5% error level, there was a statistically significant difference in the average antibacterial effect among the irrigation methods: Ultrasonic, XP Endofinisher file, and Laser Diode 810 nm, as well as when compared to the control group.

**Table 3:** Comparison of the average antibacterial effect in Ultrasonic, XP Endofinisher, and Laser irrigation methods based on Kruskal-Wallis test

Variable	Mean value	Standard deviation	Test statistic	Significance level
Ultrasonic	485.00	53.343		
XP Endofinisher	2291.67	202.072		
Laser	70.00	94.099	44.158	<0.001*
Control	4733333.3	2250387.172		

*Significance level 0.05

Next, in Table 4, multiple comparisons are given for the average antibacterial effect of Ultrasonic, XP Endofinisher, laser and the control group. 

**Table 4:** Multiple comparison of the average antibacterial effect of Ultrasonic, XP Endofinisher and Laser method

	Test statistic	Standard error	Significance level
Laser-Ultrasonic	12.00	5.711	0.036*
Laser-XP Endofinisher	-24.00	5.711	<0.001*
Laser-Control	36.00	5.711	<0.001*
Ultrasonic- XP Endofinisher	-12.00	5.711	0.036*
Ultrasonic-Control	24.00	5.711	<0.001*
XP Endofinisher-Control	12.00	5.711	0.036*

*Significance level 0.05

## Discussion

*E. faecalis* plays a significant role in primary and secondary endodontic infections, and it is not completely eliminated in root canal treatments [ 18 ]. Therefore, in this study the antimicrobial effect of three methods including PUI (Ultra X), irrigating through XP Endofinisher file and Laser Diode 810 nm on these bacteria have been compared. Accordingly, it was tried to use single-rooted teeth with the same root volume, because the effect of irrigating agents depends on the root volume [ 18 ].

Therefore, the antibacterial effect of these three methods has been determined by comparing the reduction of CFU of *E. faecalis* bacteria, which is considered as an accepted method for evaluating the antimicrobial effect [ 19 ]. The volume of the irrigating agent and the penetration rate of the instrument into the root canal can be effective in the disinfection power. Hence, we used the same volume of the irrigation agent for all three groups and the penetrate-on rate of the instrument was determined up to one millimeter of working length [ 18 ]. Moreover, the irrigation time is another effective factor, which in the present study; the groups were irrigated for the same time [ 20 ].

This study showed that all three irrigation methods including Ultra X, XP Endofinisher file and Laser Diode 810nm were effective in the reduction of *E. faecalis* CFU, which was significant compared to the control group. Furthermore, the Laser Diode 810nm was more efficient in reducing the number of bacteria in comparison with the other two methods and this difference was significant. By comparing Ultra X and XP Endofinisher irrigation methods, data showed that Ultra X had a greater reduction in CFU of bacteria and this difference was significant compared to the XP EndoFinisher group.

The Ultra X instrument uses passive ultrasonic technology and a frequency of 25-40kH by creating an eddy flow that causes the irrigation solution to agitate inside the root canal and the irrigating agent to flow to areas with difficult access in the root canal system [ 21 , 11 ]. The Ultra X handpiece has two selectable powers, and we applied its high power in this study [ 11 ]. 

The XP Endofinisher is a nickel-titanium rotary file characterized by its zero taper and unique alloy composition, which allows it to effectively clean inaccessible areas of the root canal without altering the original shape of the canal [ 22 ]. Carvalho *et al*. [ 23 ] concluded that the XP Endofinisher file as a method of activating the irrigation solution can reduce the number of *E. faecalis* bacteria in the oval canals, which is in line with the results of this study. 

The introduction of lasers in endodontics has dramatically improved the effectiveness and success rate of root canal treatment [ 24 ]. In recent years, the use of the Erbium lasers (Er: YAG, 2940 nm; Er, Cr: YSGG, 2790 nm) for the agitation of intra-canal water-based fluids has been popularized due to its absorption in water and hydroxyapatite [ 25 ]. In addition, they are more efficient in elimination of the intra-canal smear layer and biofilms, but their bactericidal effect is superficial. One of the limitations of this laser is the probability thermal damage of periradicular tissues through the open apical foramen occurring during the usage of the erbium lasers at ablative settings [ 24 ].

Arslan *et al*. [ 26 ] confirmed that the Er:YAG laser was more effective in removing superficial debris across all thirds of the root canal compared to traditional methods, while the diode laser demonstrated superior efficacy in the middle and apical thirds. Both lasers outperformed NaOCl and citric acid in terms of debris removal. Er:YAG laser irradiation is absorbed by the water, and it ablates the hard tissues. However, the irradiation by the diode laser is poorly absorbed by the hard tissues. In the study conducted by Dhawan *et al*. [ 27 ], smear layer removal efficacy of Er:YAG laser was more at coronal, middle, and apical third compared to diode laser.

The Nd:YAG laser (1064 nm) is another effective tool for disinfecting root canals, primarily utilizing thermal heating to exert its antimicrobial effects on the bacterial environment. It exhibits a well-documented absorption profile, particularly in melanin and dark pigmented tissues, which makes it effective for certain applications in medical and dental fields. However, it has poor absorption in water, which limits its effectiveness against non-pigmented bacteria such as *E. faecalis* [ 28 ].

Research show that CO_2_ laser irradiation has antibacterial effects on bacteria that are embedded in biofilm due to its photothermal mechanism [ 29 ]. Studies show that the 9, 300-nm wavelength can ablate dental soft and hard tissues and also can strongly absorb in hydroxyapatite and water. So, it can be suitable for the laser activated irrigation technique [ 30 , 31 ]. 

Diode lasers, particularly those operating at 810 nm, are increasingly utilized in endodontic treatments for photothermal disinfection and activation of irrigants such as NaOCl and EDTA. Ashofteh *et al*. [ 32 ] reported a 97.56% reduction in the number of bacteria with using diode laser and claimed that this laser can be considered as an alternative technique for root canal disinfection. Its small size, flexible and thin fiber, allows for easy access to narrow canals, enhancing the efficacy of disinfection in radicular dentinal tubules [ 33 - 34 ] while other lasers, such as the Er:YAG laser, provide better disinfection, we chose to use the diode laser in the present study due to its reported benefits in many studies, as well as its reasonable price and cost-effectiveness in clinical use

The mechanism of the Laser Diode 810 nm effect is that the laser beam penetrates into dentin and dentinal tubules which causes changes in the biological structures of bacteria and exerts its bactericidal function through major changes in the bacterial wall [ 35 ]. Asnaashari *et al*. [ 36 ] showed that irradiation of both 810 and 980 nm lasers significantly decreased the *E. faecalis* count in the root canal system, but the 810 nm laser was more effective in decreasing the intracanal microbial load. These results align with *Beer* *et al*. [ 37 ] study who demonstrated that both 810 nm and 940 nm wavelengths of laser significantly reduced colony counts of *E. faecalis* and *Escherichia coli* (*E. coli*) by approximately 98% when the access cavity was included in the irradiation process. However, the efficacy of both laser wavelengths in reducing microorganisms was nearly identical, with the 810 nm laser showing slightly greater effectiveness based on the advantages of the 810 nm laser, we used this laser in our research.

​Alakshar *et al*. [ 38 ] investigated the effect of XP Endo finisher file in removing the smear layer and debris with the Endoctivator instrument through a scanning electron microscope. The obtained results showed that the Endoactivator had a better performance compared to the XP Endofinisher file in removing debris and smear layer and this difference in the middle and apical thirds of the root canal was significant.

Espinoza *et al*. [ 39 ] investigated the effect of two irrigation methods, XP Endofinisher file and passive ultrasonic in removing smear layer and debris. They observed that the effect of passive ultrasonic in removing debris and smear layer is greater than the XP Endofinisher file and this difference is significant. In our study, the irrigation method with Ultra X (passive ultrasonic) was significantly more effective in reducing *E. faecalis* bacteria than the XP Endofinisher file, which is similar to the results of Espinoza's study [ 39 ]. Also, Bisen *et al*. [ 40 ] compared the effectiveness of the XP Endofinisher file and passive ultrasonic in smear layer removal. Their results showed that there was no difference between these two methods in the coronal third and the middle third of the root canal, but the XP Endofinisher file was more effective in the apical third, which contrasts with the obtained results from Espinoza *et al*. [ 39 ] and the present study. The XP Endofinisher file has a unique cycle-like shape that allows it to maintain better contact with the canal wall in the apical third of narrow canals. This design feature enhances its ability to clean and disinfect these challenging areas effectively.

One of the limitations of this study was related to sample collection. The objective was to find single-canal teeth samples that were similar in size and shape, which could culture *E. faecalis* and undergo the irrigation process consistently. However, it proved difficult to locate teeth meeting these specific criteria, which may have impacted the study's overall findings and applicability. 

## Conclusion

Based on the results of this study, all three methods- PUI, XP Endofinisher file, and Laser Diode 810nm- were effective in reducing bacteria within the root canal. Notably, the antibacterial effect of the Laser Diode 810nm was found to be more efficient than that of the other two irrigation methods, including PUI and XP Endofinisher. Therefore, to determine the standard irrigation method, more studies are needed in this field along with the comparison of different irrigation methods with each other.
